# Polyphenols and Triterpenes Combination in an In Vitro Model of Cardiac Damage: Protective Effects

**DOI:** 10.3390/ijms24097977

**Published:** 2023-04-28

**Authors:** Natalia de las Heras, Adrián Galiana, Sandra Ballesteros, José Carlos Quintela, Ileana Bonilauri, Vicente Lahera, Beatriz Martín-Fernández

**Affiliations:** 1 Department of Physiology, Faculty of Medicine, Plaza Ramón y Cajal, s/n, Universidad Complutense, 28040 Madrid, Spain; nlashera@ucm.es (N.d.l.H.); adrian.galiana@udima.es (A.G.); sballest@ucm.es (S.B.); vlahera@ucm.es (V.L.); 2 Scientific Department, Natac Biotech, 28923 Madrid, Spain; jcquintela@natacgroup.com; 3 Department of Molecular Biology, Faculty of Biology, Universidad de León, Campus de Vegazana s/n, 24071 León, Spain; ibonil00@estudiantes.unileon.es

**Keywords:** polyphenols, triterpenes, cardiac cell viability, oxidative stress, inflammation

## Abstract

Olive products contain high levels of monounsaturated fatty acids as well as other minor components such as triterpenic alcohols and other pentacyclic triterpenes, which together form the main triterpenes of virgin olive oil. Olive fruits and leaves contain significant amounts of hydrophilic and lipophilic bioactives including flavones, phenolic acids and phenolic alcohols, amongst others. Several studies have shown the benefits of these substances on the cardiovascular system. Regardless, little is known about the specific combination of bioactive compounds in cardiovascular health. Thus, we aimed to test the combination of a triterpenes (TT70) and a polyphenols (HT60) olive oil bioactive extract in H9c2 cells under stress conditions: LPS and H_2_O_2_ stimulation. To evaluate the effectiveness of the combination, we measured cell viability, superoxide production and protein expression of caspase 3, eNOS, peNOS, TNF-α and Il-6. Overall, cells stimulated with LPS or H_2_O_2_ and co-incubated with the combination of triterpenes and polyphenols had increased cell survival, lower levels of superoxide anion, lower protein expression of eNOS and higher expression of peNOS, increased protein expression of SOD-1 and lower protein expression of TNF-α and Il-6. The specific combination of HT60+TT70 is of great interest for further study as a possible treatment for cardiovascular damage.

## 1. Introduction

The so-called Mediterranean diet, which is known to be among the healthiest, consists mainly of vegetables, fruits, cereals, rice, pasta and bread, and olive oil is the main source of fat [1,2]. Most of the available data supporting the beneficial effect of the Mediterranean diet on health derive from studies linking diet and cardiovascular disease, the leading cause of morbidity and mortality worldwide.

The PREDIMED study demonstrated that an energy-unrestricted Mediterranean diet supplemented with either extra-virgin olive oil or nuts reduced the absolute risk of approximately three major cardiovascular events per 1000 person-years, for a relative risk reduction of approximately 30%, among high-risk persons who were initially free of cardiovascular disease. The results obtained support the benefits of the Mediterranean diet in reducing cardiovascular risk and metabolic syndrome [1]. Several studies have shown that the consumption of virgin olive oil reduces blood pressure values and cholesterol levels, as well as other risk factors involved in cardiovascular disease [3,4]. In fact, greater adherence to the Mediterranean diet was associated with a lower risk of general and cardiovascular mortality, as shown in a meta-analysis involving more than 1.5 million healthy subjects followed up for between 3 and 18 years and 40,000 fatal and non-fatal events [5]. 

Olive products contain high levels of monounsaturated fatty acids (MUFAs) as well as other minor components [6]. Around 98% of the chemical composition of the oil is made up of triacylglycerides, partial glycerides, esters of fatty acids or free fatty acids and phosphatides, which represent the saponifiable fraction of olive oil. In the unsaponifiable fraction of virgin olive oil, there are triterpenic alcohols and other pentacyclic triterpenes, which together form the main triterpenes of virgin olive oil [7,8]. Oleanolic acid, maslinic acid, uvaol and erythrodiol are the main triterpenes in virgin olive oil [8,9]. It has been described that the activity of triterpenes in virgin olive oil has bioactive properties such as antitumor, anti-inflammatory, antioxidant, hepatoprotective, cardioprotective and antimicrobial activity [8]. As a result of all these actions, phytogenics derived from the olive tree (*Olea europaea* L.) have been shown to be beneficial for several diseases such as metabolic syndrome or coronary heart disease, with a high incidence in the population [10].

On the other hand, olive fruits and leaves contain significant amounts of hydrophilic and lipophilic bioactives including flavones, phenolic acids, phenolic alcohols, secoiridoids and hydroxycinnamic acid derivates [11]. Olive oil polyphenols contribute to the protection of blood lipids from oxidative stress. As a result of their anti-inflammatory, antioxidant, and antimicrobial actions, olive-derived plant bioactives have been shown to cause several beneficial effects under pathological conditions [12,13,14], which renders them promising in terms of beneficial effects on cardiovascular health.

Taking into account the beneficial effects of triterpenes and polyphenols individually, the idea of combining both bioactive compounds to provide additional positive effects on cardiovascular protection arises. In this sense, previous studies have shown in pigs [15] and fish [16] that a bioactive extract of olive oil, which contains a mixture of triterpenic acid and polyphenols, had anti-inflammatory and immunomodulatory properties in the intestine, while it also improved the integrity of the epithelium. Furthermore, a recent study showed that these compounds could reduce systemic inflammation in cattle [17].

Several studies have shown the benefits of these substances on the cardiovascular system, on the nervous system, and on cancer cells, and it is believed that this is due to their antioxidative, anti-inflammatory, anti-mutagenic, and anti-carcinogenic properties, in addition to the modulation of enzymatic functions [18,19,20,21]. A higher consumption of flavonoid-rich foods is linked to a reduction in cardiovascular morbidity and mortality according to various epidemiological studies [22,23]. Regardless of these results, little is known about the specific combination of bioactive compounds in cardiovascular health.

Considering the beneficial effects of both metabolites, our study hypothesizes about the possible synergistic effect that a combination of both substances could have, providing a greater cardioprotective effect than individually administered. There is an interplay between oxidative stress and inflammatory molecules that generates an environment prone to cardiac damage. Thus, we aimed to test the combination of a triterpenes and a polyphenols olive oil bioactive extract in rat cardiac cells under stress conditions: lipopolysaccharide (LPS) and hydrogen peroxide (H_2_O_2_) stimulation. To evaluate the effectiveness of the combination, we measured cell viability, superoxide production and protein expression of caspase 3, endothelial nitric oxide synthase (eNOS), phosphorylated endothelial nitric oxide synthase (peNOS), tumor necrosis factor alpha (TNF-α) and interleukin 6 (Il-6).

## 2. Results

### 2.1. Cell Viability and Apoptosis

#### 2.1.1. Cell Viability 

**Figure 1:**
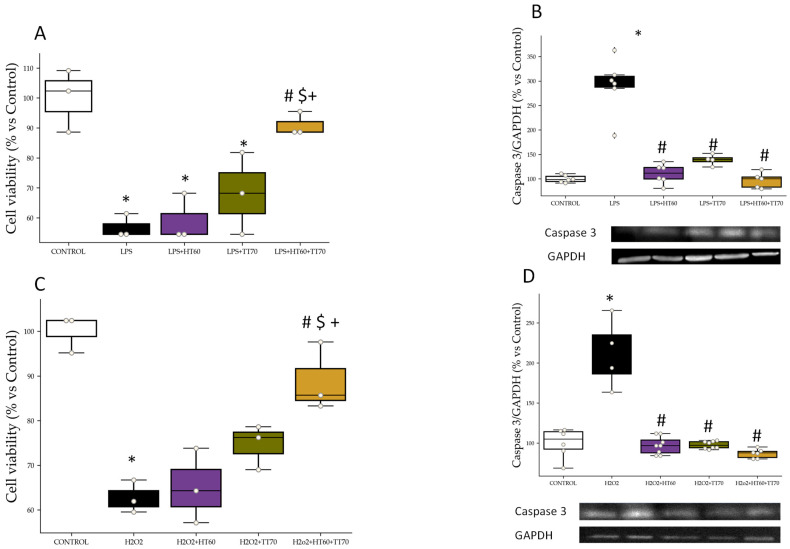
HT60, TT70 and HT60+TT70 effects on cell viability in H9c2 cells stimulated with LPS or H_2_O_2_. (**A**) Cell viability of LPS-stimulated cells. (**B**) Relative protein expression levels by western blot of caspase 3 in LPS-stimulated cells. (**C**) Cell viability of H_2_O_2_-stimulated cells. (**D**) Relative protein expression levels by western blot of caspase 3 in H_2_O_2_-stimulated cells. Data are expressed as percentage of mean ± SEM versus CONTROL group. * *p* < 0.05 vs. CONTROL; # *p* < 0.05 vs. LPS or H_2_O_2;_ $ *p* < 0.05 vs. HT60; + *p* < 0.05 vs. TT70. CONTROL: Control group; LPS: lipopolysaccharide group; H_2_O_2_: hydrogen peroxide group; LPS+HT60: LPS+polyphenols extract group; LPS+TT70: LPS+triterpenes extract group; LPS+HT60+TT70: LPS+polyphneols+triterpenes group; H_2_O_2_+HT60: H_2_O_2_+polyphenols extract group; H_2_O_2_+TT70: H_2_O_2_+triterpenes extract group; H_2_O_2_+HT60+TT70: H_2_O_2_+polyphneols+triterpenes group.

Cell viability decreased in LPS-Stimulated Cells (*p* < 0.01) Compared with CONTROL Group. Cells stimulated with LPS and co-incubated with HT60 or TT70 alone showed decreased cell viability compared with CONTROL (*p* < 0.05) and no significant differences compared with LPS. On the other hand, co-incubation with HT60+TT70 combination of LPS-stimulated cells was able to prevent cell viability descending (*p* < 0.01) as shown in Figure 1A.

Regarding H_2_O_2_ stimulation, stimulated cells (*p* < 0.01) showed decreased viability compared with CONTROL group. Cells stimulated with H_2_O_2_ and co-incubated with HT60 or TT70 alone showed decreased cell viability compared with CONTROL (*p* < 0.05) and no significant differences compared with H_2_O. On the other hand, co-incubation with HT60+TT70 combination of H_2_O_2_-stimulated cells was able to prevent cell death (*p* < 0.01) as shown in Figure 1C.

#### 2.1.2. Caspase 3 Protein Expression

Caspase 3 levels were increased (*p* < 0.05) in LPS-stimulated cells compared with CONTROL (Figure 1B). Co-incubation of LPS-stimulated cells with HT60 or TT70 decreased (*p* < 0.05) caspase 3 levels compared with LPS (Figure 1B). HT60+TT70 combination decreased caspase 3 to a greater extent than individual treatments and significant differences (*p* < 0.05) were found compared to TT70 condition (Figure 1B).

Caspase 3 levels increased (*p* < 0.05) in H_2_O_2_-stimulated cells compared with CONTROL (Figure 1D). Co-incubation of H_2_O_2_-stimulated cells with HT60 or TT70 and combination HT60+TT70 decreased (*p* < 0.05) caspase 3 levels compared with LPS to a similar extent (Figure 1D).

### 2.2. Oxidative Stress Status

#### 2.2.1. Superoxide Detection

**Figure 2:**
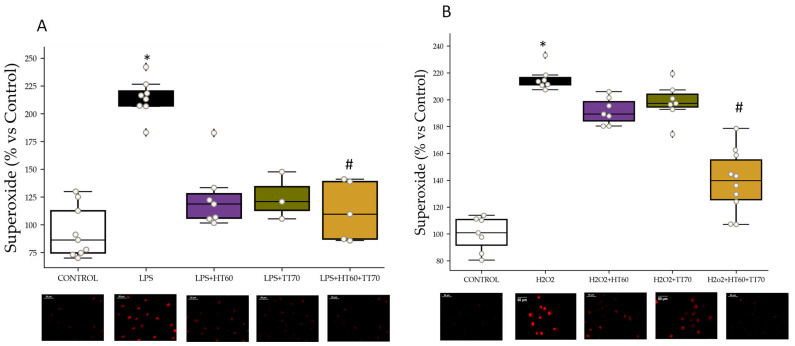
HT60, TT70 and HT60+TT70 effects on superoxide production in H9c2 cells stimulated with LPS or H_2_O_2_ and representative microphotographs of each condition. (**A**) Superoxide production in LPS-stimulated cells. (**B**) Superoxide production in H_2_O_2_-stimulated cells. Data are expressed as percentage of mean ± SEM versus CONTROL group. * *p* < 0.05 vs. CONTROL; # *p* < 0.05 vs. LPS or H_2_O_2_. CONTROL: Control group; LPS: lipopolysaccharide group; H_2_O_2_: hydrogen peroxide group; LPS+HT60: LPS+polyphenols extract group; LPS+TT70: LPS+triterpenes extract group; LPS+HT60+TT70: LPS+polyphneols+triterpenes group; H_2_O_2_+HT60: H_2_O_2_+polyphenols extract group; H_2_O_2_+TT70: H_2_O_2_+triterpenes extract group; H_2_O_2_+HT60+TT70: H_2_O_2_+polyphneols+triterpenes group.

Superoxide detection increased in LPS-stimulated cells (*p* < 0.01) compared to CONTROL. Cells stimulated with LPS and co-incubated with HT60 or TT70 alone increased superoxide levels compared with CONTROL (*p* < 0.05) and decreased compared with LPS (*p* < 0.05). Co-incubation with HT60+TT70 combination of LPS-stimulated cells prevented superoxide increase (*p* < 0.01) compared with LPS as shown in Figure 2A.

H_2_O_2-_stimulated cells (*p* < 0.01) showed highly increased superoxide detection compared with CONTROL group. Cells stimulated with H_2_O_2_ and co-incubated with HT60 or TT70 alone increased superoxide levels compared with CONTROL (*p* < 0.05) and decreased compared with H_2_O_2_ (*p* < 0.05). On the other hand, co-incubation with HT60+TT70 combination of H_2_O_2_-stimulated cells prevented superoxide increase (*p* < 0.05) compared with H_2_O_2_ and with individual treatments (*p* < 0.05) as shown in Figure 2B.

#### 2.2.2. eNOS, peNOS Protein Expression and peNOS/eNOS Ratio

**Figure 3:**
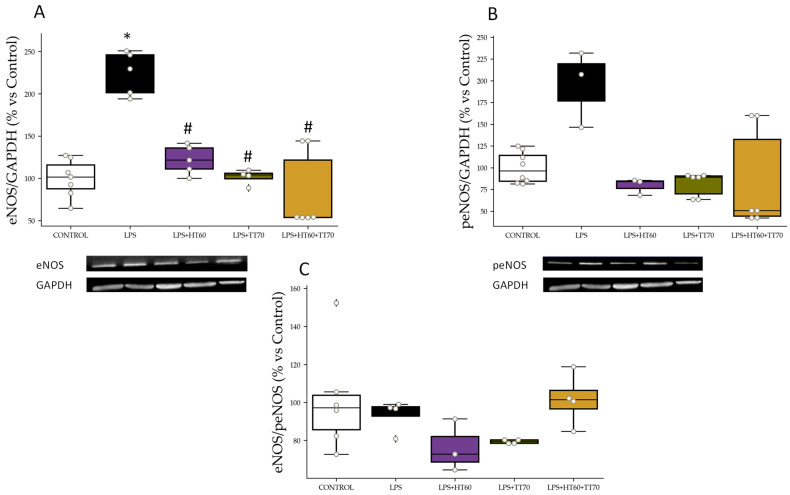
HT60, TT70 and HT60+TT70 effects on eNOS and peNOS protein expression and eNOS/peNOS ratio in H9c2 cells stimulated with LPS. (**A**) Relative protein expression levels by western blot of eNOS in LPS-stimulated cells. (**B**) Relative protein expression levels by western blot of peNOS in LPS-stimulated cells. (**C**) eNOS/peNOS ratio of LPS-stimulated cells. Data are expressed as percentage of mean ± SEM versus CONTROL group. * *p* < 0.05 vs. CONTROL; # *p* < 0.05 vs. LPS. CONTROL: Control group; H_2_O_2_: hydrogen peroxide group; LPS+HT60: LPS+polyphenols extract group; LPS+TT70: LPS+triterpenes extract group; LPS+HT60+TT70: LPS+polyphneols+triterpenes group.

eNOS levels were higher (*p* < 0.05) in LPS-stimulated cells compared with CONTROL (Figure 3A). Similarly, peNOS levels were higher (*p* < 0.05) in LPS-stimulated cells compared with CONTROL and reversed (*p* < 0.05) by co-incubation with each treatment, alone or combined (Figure 3B). No significant differences were observed amongst groups in peNOS/eNOS ratio (Figure 3C).

**Figure 4:**
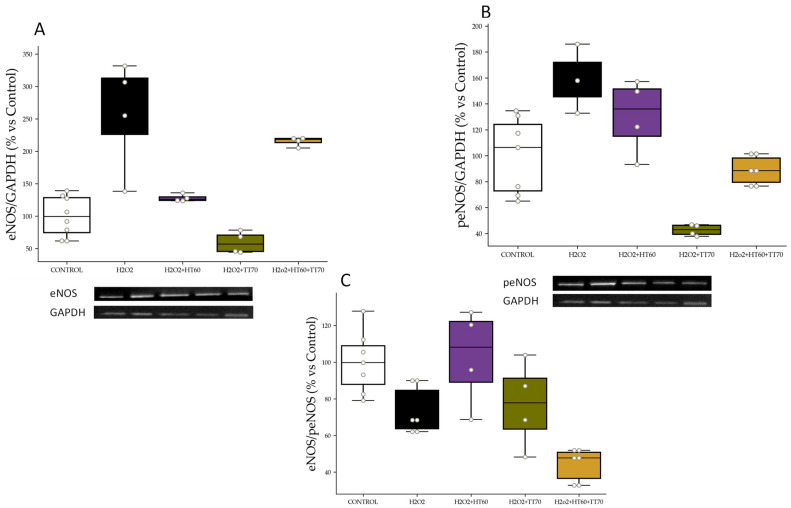
HT60, TT70 and HT60+TT70 effects on eNOS and peNOS protein expression and eNOS/peNOS ratio in H9c2 cells stimulated with H_2_O_2_. (**A**) Relative protein expression levels by western blot of eNOS in H_2_O_2_-stimulated cells. (**B**) Relative protein expression levels by western blot of peNOS in H_2_O_2_-stimulated cells. (**C**) eNOS/peNOS ratio of H_2_O_2_-stimulated cells. Data are expressed as percentage of mean ± SEM versus CONTROL group. CONTROL: Control group; H_2_O_2_: hydrogen peroxide group; H_2_O_2_+HT60: H_2_O_2_+polyphenols extract group; H_2_O_2_+TT70: H_2_O_2_+triterpenes extract group; H_2_O_2_+HT60+TT70: H_2_O_2_+polyphneols+triterpenes group.

eNOS levels were higher (*p* < 0.05) in H_2_O_2_-stimulated cells compared with CONTROL which was reversed (*p* < 0.05) by co-incubation with HT60 and TT70 individually (Figure 4A). HT60+TT70 combination increased (*p* < 0.05) eNOS levels when compared to H_2_O_2_-stimulated cells and co-incubated with individual HT 60 or TT70 (Figure 4A). peNOS levels were higher (*p* < 0.05) in H_2_O_2_-stimulated cells compared with CONTROL and lower in H_2_O_2_-stimulated cells co-incubated with individual or combined extracts (Figure 4B). peNOS/eNOS ratio decreased (*p* < 0.05) in H_2_O_2_-stimulated cells compared with CONTROL (Figure 4C). No significant differences were observed in the co-incubation with individual extracts, but HT60+TT70 combination showed decreased (*p* < 0.05) peNOS/eNOS ratio compared with the rest of the groups (Figure 4C).

### 2.3. SOD1 Protein Expression

**Figure 5:**
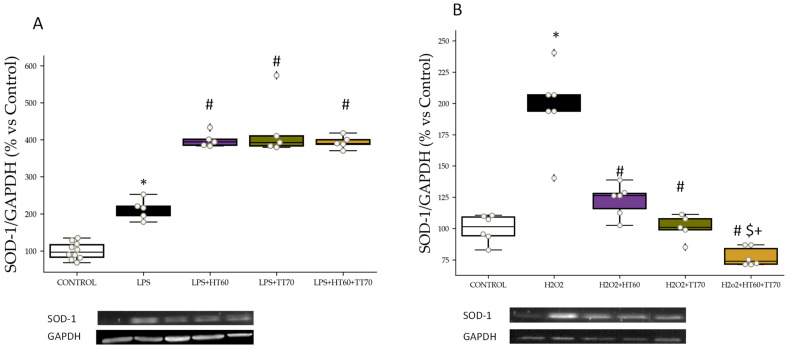
HT60, TT70 and HT60+TT70 effects on SOD-1 protein expression in H9c2 cells stimulated with LPS or H_2_O_2_. (**A**) Relative protein expression levels by western blot of SOD-1 in LPS-stimulated cells. (**B**) Relative protein expression levels by western blot of SOD-1 in H_2_O_2_-stimulated cells. Data are expressed as percentage of mean ± SEM versus CONTROL group. * *p* < 0.05 vs. CONTROL; # *p* < 0.05 vs. LPS or H_2_O_2_; $ *p* < 0.05 vs. HT60; + *p* < 0.05 vs. TT70. CONTROL: Control group; LPS: lipopolysaccharide group; H_2_O_2_: hydrogen peroxide group; LPS+HT60: LPS+polyphenols extract group; LPS+TT70: LPS+triterpenes extract group; LPS+HT60+TT70: LPS+polyphneols+triterpenes group; H_2_O_2_+HT60: H_2_O_2_+polyphenols extract group; H_2_O_2_+TT70: H_2_O_2_+triterpenes extract group; H_2_O_2_+HT60+TT70: H_2_O_2_+polyphneols+triterpenes group.

SOD1 protein expression was increased (*p* < 0.05) in LPS-stimulated cells compared with CONTROL (Figure 5A). Co-incubation of LPS-stimulated cells with individual extracts, HT60 and TT70, or combined HT60+TT70, increased (*p* < 0.05) SOD1 levels compared with CONTROL and LPS (Figure 5A).

No significant differences were observed in H_2_O_2_-stimulated cells compared with CONTROL (Figure 5B). Co-incubation with TT70 decreased SOD1 levels compared with H_2_O_2_ group and co-incubation with HT60+TT70 combination increased (*p* < 0.05) SOD1 levels compared with TT70 individual extract (Figure 5B).

### 2.4. Inflammatory Markers: TNF-α and Il-6

**Figure 6:**
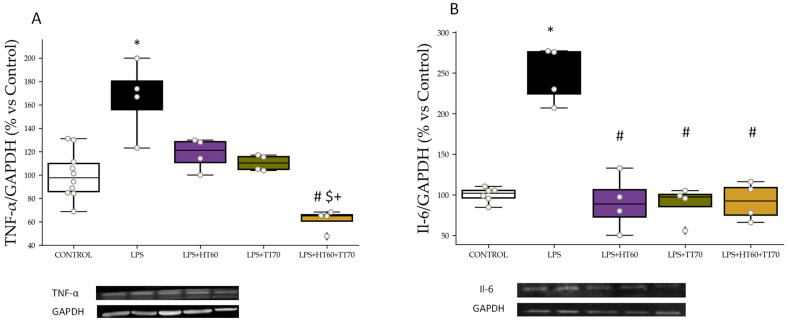
HT60, TT70 and HT60+TT70 effects on TNF-α and Il-6 in H9c2 cells stimulated with LPS. (**A**) Relative protein expression levels by western blot of TNF-α in LPS-stimulated cells. (**B**) Relative protein expression levels by western blot of Il-6 in LPS-stimulated cells. Data are expressed as percentage of mean ± SEM versus CONTROL group. * *p* < 0.05 vs. CONTROL; # *p* < 0.05 vs. LPS; $ *p* < 0.05 vs. HT60; + *p* < 0.05 vs. TT70. CONTROL: Control group; LPS: lipopolysaccharide group; LPS+HT60: LPS+polyphenols extract group; LPS+TT70: LPS+triterpenes extract group; LPS+HT60+TT70: LPS+polyphneols+triterpenes group.

TNF-α levels increased (*p* < 0.01) in LPS-stimulated cells compared with CONTROL (Figure 6A), whereas no significant differences were observed in individual LPS+HT60 or TT70 co-incubation when compared with LPS-stimulated cells. On the other hand, co-incubation with HT60+TT70 combination decreased (*p* < 0.05) TNF-α levels compared with LPS group and individual extracts (Figure 6A).

Il-6 levels increased (*p* < 0.05) in LPS-stimulated cells compared with CONTROL (Figure 6A). Co-incubation of LPS-stimulated cells with HT60, TT70 or HT60+TT70 decreased (*p* < 0.05) Il-6 protein expression compared with LPS to a similar extent (Figure 6B).

**Figure 7:**
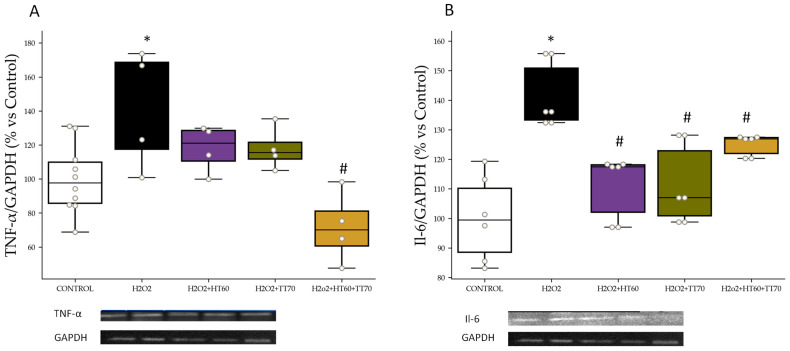
HT60, TT70 and HT60+TT70 effects on TNF-α and Il-6 in H9c2 cells stimulated with H_2_O_2_. (**A**) Relative protein expression levels by western blot of TNF-α in H_2_O_2_-stimulated cells. (**B**) Relative protein expression levels by western blot of Il-6 in H_2_O_2_-stimulated cells. Data are expressed as percentage of mean ± SEM versus CONTROL group. * *p* < 0.05 vs. CONTROL; # *p* < 0.05 vs. H_2_O_2_. CONTROL: Control group; H_2_O_2_: hydrogen peroxide group; H_2_O_2_+HT60: H_2_O_2_+polyphenols extract group; H_2_O_2_+TT70: H_2_O_2_+triterpenes extract group; H_2_O_2_+HT60+TT70: H_2_O_2_+polyphneols+triterpenes group.

Regarding H_2_O_2_, TNF-α levels increased (*p* < 0.05) in H_2_O_2_-stimulated cells compared with CONTROL (Figure 7A), whereas no significant differences were observed in individual H_2_O_2_+HT60 or TT70 co-incubation when compared with H_2_O_2_-stimulated cells. On the other hand, co-incubation with HT60+TT70 combination decreased TNF-α levels compared with H_2_O_2_ group and individual extracts (Figure 7A).

Il-6 levels were increased (*p* < 0.05) in H_2_O_2_-stimulated cells compared with CONTROL (Figure 7A). Co-incubation of H_2_O_2_-stimulated cells with HT60, TT70 or HT60+TT70 decreased (*p* < 0.05) Il-6 compared with H_2_O_2_ to a similar extent (Figure 7B).

### 2.5. Statistical Analysis

In this experiment, we used python 3.7.6 language libraries for data processing. Statistical tests of normality (Shapiro–Wilk) and significance (one-way ANOVA) for the studied variables were performed. Tukey’s test was used for post hoc analyses. The data were represented in boxplots showing median, first and third quartile, and minimum and maximum values; the dots show individual values. The level of significance was set at *p* < 0.05. 

## 3. Discussion

In the present study, we have observed that, when combined, triterpenes and polyphenols confer greater protection against cell damage than individually in cells stimulated with LPS or H_2_O_2_. Overall, cells stimulated with LPS or H_2_O_2_ and co-incubated with the combination of triterpenes and polyphenols had increased cell survival, lower levels of superoxide anion, lower protein expression of eNOS and higher expression of peNOS, increased protein expression of SOD-1 and lower protein expression of TNF-α and Il-6.

### 3.1. HT60+TT70 Prevents Cell Loss under Stress Conditions in H9c2 Cells; ROS-CaspasesPathway

As previously described, cells under stress conditions stimulated with LPS or H_2_O_2_ showed conservated cell viability when co-incubated with HT60+TT70 and to a greater extent than cells co-incubated with individual extracts. Cell death was stimulated by stressors such as those mediated by various factors and mechanisms, including oxidative stress. Oxidative stress is a hallmark of cardiovascular disease and constitutes a major mechanism of many cardiovascular pathophysiologies [24]. Classic ROS (reactive oxygen species)-mediated mechanism of lysosomal membrane permeabilization leads to lysosomal-dependent cell death. ROS can trigger many components of the apoptotic machinery, including activating mitogen-activated protein kinases like c-Jun N-terminal kinase (JNK) and extracellular signal-regulated kinase, activating caspases and their downstream effectors like poly ADP-ribose polymerase, and increasing expression of pro-apoptotic mitochondrial factors like Bcl and Bax [25]. In our work, we have observed exacerbated superoxide production in cells stimulated with LPS or H_2_O_2_ and, in turn, a loss in the balance of protein expression of eNOS and peNOS. Pathological conditions, such as hypertension and other cardiovascular risk factors, uncoupling of eNOS takes place contributing to reduction in nitric oxide (NO) production but also to an increase of NO inactivation by ROS such as superoxide anion [26,27]. We have described in previous studies antihypertensive effects together with vascular and hypertension target organ protection in spontaneously hypertensive rats together with enhanced protein expression of eNOS of a pomace oil extract [4]. Here, we are observing that the combination of polyphenols and triterpenes are conferring bigger protection against uncoupling of eNOS than individual administration. In this sense, the main bioactive molecule in TT70, oleanolic acid, might be enhancing antioxidant properties of hydroxytyrosol, highly concentrated in HT60. Studies conducted on neuronal and hepatic damage models have shown certain antioxidant effects of oleanolic acid [28,29]. The triterpene extract combined with hydroxytyrosol could provide a strong capacity to decrease oxidative stress. Nevertheless, further studies are necessary to confirm this hypothesis, even though the results suggest the two bioactive molecules could be acting in a synergic way. 

As mentioned, ROS induces caspase activation under stress conditions. Here, we observed increased caspase 3 relative protein expression in cells stimulated with LPS or H_2_O_2_ which emphasizes the role of ROS as one of the main mechanisms involved in the decreased cell viability observed in our study. On the contrary, the combination of HT60+TT70 decreased caspase 3 protein expression compared with stimuli, especially H_2_O_2_, and to a greater extent, compared to individual extracts’ co-incubation_._ Previous studies have shown the effects of polyphenols modulating caspase 3 activation [25,30]. Triterpenes have also been studied as antiapoptotic metabolites since they show some modest effects on death cell mediators [31,32]. However, in our study, we are observing a bigger effect of the molecules when they are administered combined than alone. In an animal production context, it has been observed that a combination of the polyphenol verbascoside and triterpenic compounds provided host’s immunity and enhanced disease resistance [33]. These results suggest that the co-combination of both bioactive molecules may have a very relevant role in human health, particularly cardiovascular health.

### 3.2. HT60+TT70 as a Potent Antioxidant Combination in Cardiac Damage

The LPS group showed increased cardiac antioxidant capacity by stimulating protein expression of SOD1. Antioxidant defense mechanisms, such as synthesis of the detoxification enzymes SOD1, catalase, glutathione S-transferase, glutathione peroxidase and NADPH induction, have been proposed as one of the main mechanisms of action of polyphenols [34,35,36]. In addition, a study conducted in H9c2 cells has shown treatment with escin, pentacyclic triterpenoid saponin, greatly increased the expression levels of SOD1 and SOD2 [37]. Co-incubation with individual and combined extracts showed increased SOD1 compared with Control and LPS showing off the great antioxidant capacity of both extracts. Nevertheless, when cells were stimulated with potent oxidant agent H_2_O_2_, the individual triterpenes extract was not able to increase the antioxidant capacity until it was combined with polyphenolic extract. Once again, the results suggest that a positive effect might exist when polyphenols and triterpenes are combined.

### 3.3. HT60+TT70 Attenuates Pro-Inflammatory Status under Stress Conditions in H9c2 Cells

It is well known that oxidative stress imbalance manifests as inflammation represented by the release of proinflammatory cytokines like TNF-α [38]. In our study, we observed increased protein expression of TNF-α and Il-6 in H9c2 cells under stress conditions, especially LPS. LPS-stimulated production of cytokines, such as TNF-α, IL-6, IL-10 and IFN-γ can induce apoptosis of cardiomyocytes [39,40,41]. Individual treatments reduced protein expression of both pro-inflammatory markers, but once again, the combination of HT60+TT70 was able to normalize the data. Oleanolic acid has been described as a potent anti-inflammatory agent in cardiovascular risk situations such as obesity [29]. In addition, maslinic acid, the second majoritarian bioactive compound in TT70, has shown anti-inflammatory effects by NF-κB and STAT-1 in umbilical vein endothelial cells treated with LPS [42]. On the other hand, polyphenols have been shown to modulate inflammatory cytokines such as TNF-α, Il-6 and Il-8 and to repress the synthesis of proinflammatory mediators and adhesion molecules in numerous pathological conditions [43,44]. In a recent clinical study, IL-6 has been proposed as a potential therapeutic target in specific heart failure subpopulations [45]. Since we are observing a potent effect of HT60 and TT70 combined controlling the pro-inflammatory response induced by cardiovascular stressors, we could hypothesize about the promising use of a specific combination of polyphenols and triterpenes in treatments of cardiovascular diseases.

## 4. Materials and Methods

### 4.1. H9c2 Culture and Differentiation

H9c2(2–1) rat embryonic cardiomyoblasts were purchased from American Type Culture Collection (ATCC, ref.: CRL-1446™) and they were cultured in Dulbecco’s modified Eagle’s medium (low glucose-DMEM) supplemented with 10% fetal bovine serum (FBS), 2 mM glutamine, 100 U/mL penicillin and 100 μg/mL streptomycin at 37 °C in a humidified atmosphere of 5% CO_2_. The cell culture medium was changed every 2–3 days. To maintain the undifferentiated phenotype, cells were sub-cultured when reaching ~80% confluence.

### 4.2. LPS, H_2_O_2_ Exposition and Extracts Co-Incubation

Cardiomyoblasts were treated with the following experimental conditions: LPS (Sigma Aldrich, Madrid, Spain) 5 μg/mL, H_2_O_2_ 0.08 mM (Sigma Aldrich, Madrid, Spain), TT70 1 μg/mL of the extract (olive oil 70% triterpenes, Natac Biotech, Madrid, Spain), HT60 0.33 μg/mL of the extract (olive oil 60% hydroxytyrosol, Natac Biotech, Madrid, Spain), LPS 5 μg/mL + TT70 1 μg/mL, LPS 5 μg/mL + HT60 0.33 μg/mL, H_2_O_2_ 0.08 mM + TT70 1 μg/mL, H_2_O_2_ 0.08 mM + HT60 0.33 μg/mL, LPS 5 μg/mL + TT70 1 μg/mL + HT60 0.33 μg/mL (TT70/HT60 1:3 ratio) and H_2_O_2_ 0.08 mM + TT70 1 μg/mL+ HT60 0.33 μg/mL (TT70/HT60 1:3 ratio). Untreated cells served as control of the experiment. 

Extracts composition: TT70 1 μg/mL (olive leaf extract was obtained with ethanol extraction and quantified as 70% total triterpenes expressed as oleanolic acid, Natac Biotech, Madrid, Spain), HT60 0.33 μg/mL (olive fruit extract obtained with water extraction and quantified as 60% hydroxytyrosol, Natac Biotech, Madrid, Spain). 

**Figure 8:**
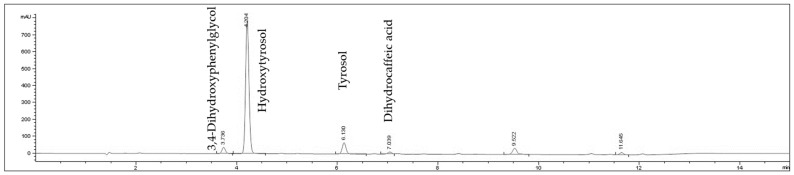
Chromatogram by HPLCUV of TT70 extract identifying the main peaks.

**Figure 9:**
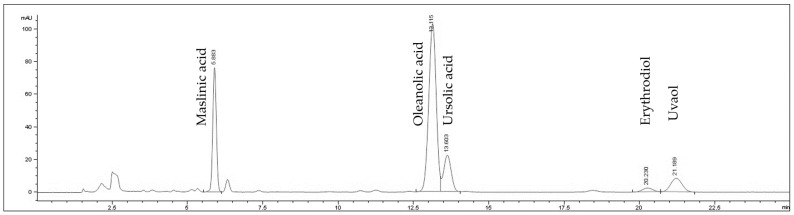
Chromatogram by HPLCUV of HT60 extract identifying the main peaks.

The extracts were analyzed by chromatogram by HPLCUV identifying the main peaks (Figure 8 and Figure 9). The TT70 extract was analyzed as described in Rada et al., 2011 [46]. The HT60 extract was analyzed using a column octadecylsilyl silica gel for chromatography; 150 mm × 4.6 mm; flow rate: 1 mL/min, wavelength 280 nm, mobile phase A: acetic acid: water (0.2:99.8 *v*/*v*); mobile phase B (methanol). Gradient min 0 (90% A); min 10 (63% A); min 11 (90% A). The identified peaks in HT-60 accounted for >93% of the total integrated area and in TT70 > 98% of the total integrated area.

### 4.3. Viability Assay

In order to assess the cytotoxicity effect of the LPS and H_2_O_2_ on cardiomyoblasts, XTT (sodium 3′-[1-(phenylaminocarbonyl)-3,4-tetrazolium]-bis (4-methoxy6-nitro) benzene sulfonic acid hydrate)) assay (In Vitro Toxicology Assay Kit, XTT based; Sigma-Aldrich, St. Louis, MO, USA) was performed in accordance with the manufacturer’s protocol. The principle of the XTT test is based on the reduction of yellow tetrazole salt by mitochondrial dehydrogenases to orange formazan, the released amount of which is directly proportional to metabolically active (living) cells.

Based on the obtained absorbance readings, the percentage of the value obtained for control cells (100%) was determined to be the calculated absorption value for the individual salinomycin concentrations. The absorbance results from untreated cells (control) were described as 100% and this value was used to calculate the absorption value for individual LPS, adalimumab concentrations. The percentage of viable cells in the culture treated with LPS, adalimumab was indicated in comparison to the untreated cells.

### 4.4. Assessment of Superoxide Anion Concentration

The production of O_2_^−^ was assessed using dihydroethidium reagent (DHE, D2307, Life Technologies, Carlsbad, CA, USA), a fluorescent dye sensitive to O_2_^−^. Twenty-four hours after aldosterone stimulation and co-incubation with the treatments, DHE was added to each well. After the incubation time had elapsed, three washes with phosphate buffer saline were performed and a minimum of eight images per experimental condition were taken with an inverted microscope (Leica DM-IL, Leica Microsystems, Buffalo Grove, IL, USA), at 40× magnification and coupled to a fluorescence unit (Leica 106Z, Leica Microsystems, Buffalo Grove, IL, USA).

### 4.5. Western Blot Analysis

To obtain purified protein, cells lysate was homogenized on ice-cold lysis buffer, containing 150 mMTris (pH 7.4), 50 mMNaCl, 1% Triton X-100, 3 mM phenylmethylsulfonyl fluoride, 3 mM dithiothreitol (Sigma, Madrid, Spain) and protease inhibitor cocktail (Roche, Munich, Germany). Proteins were separated on 15% SDS-PAGE gels under reducing conditions and transferred to nitrocellulose membranes (Bio-Rad, Hercules, CA, USA). The membranes were blocked for 1 h with 5% (*w*/*v*) BSA as blocking agent (Sigma, Madrid, Spain) in PBST (1% PBS, 0.1% Tween 20 *v*/*v*) at room temperature. After washing with PBST, the membranes were probed overnight at 4 °C with appropriate primary antibodies: anti-caspase 3 (ab4051), anti-eNOS (ab300071), anti-peNOS (ab215717), anti-SOD1 (ab308181), anti-TNFα (ab34674) and anti-Il-6 (ab233706). After washing, the membrane was incubated for 1 h with peroxidase-conjugated rabbit or mouse anti-goat IgG secondary antibody (1:10,000). For detection, ECL Advance Western Blotting Detection kit (Amersham) was used. Blots were probed with rabbit monoclonal anti-GAPDH antibody (1:10,000, Abcam, Cambridge, UK) as internal control, to normalize between gels. Quantification was expressed as percentage of relative protein expression (Protein/GAPDH) vs. CONTROL group.

## 5. Conclusions

Considering the above results, we could conclude that the combination of two bioactive molecules such as polyphenols and triterpenes prevents the loss of cell viability that occurs in situations of cardiovascular damage.

Likewise, comparing the results obtained with the individual treatments, it could be proposed that there is a synergic action between both metabolites that confers greater protective capacity than when they are administered independently. Nevertheless, further studies are needed to deepen into the possible synergistic mechanisms and to confirm the hypothesis suggested by our results.

Thus, the specific combination of HT60+TT70 is of great interest for further study as a possible treatment for cardiovascular damage.

## Data Availability

The data presented in this study are available upon request from the corresponding authors.
